# Communications between macrophages and cardiomyocytes

**DOI:** 10.1186/s12964-023-01202-4

**Published:** 2023-08-16

**Authors:** Pengbo Yang, Ziwei Chen, Wei Huang, Junhua Zhang, Lihui Zou, Haiyan Wang

**Affiliations:** 1 Department of Laboratory Medicine, Beijing Hospital, National Center of Gerontology, Institute of Geriatric Medicine, Chinese Academy of Medical Sciences, Beijing, China; 2 The Key Laboratory of Geriatrics, Beijing Institute of Geriatrics, Institute of Geriatric Medicine, Chinese Academy of Medical Sciences, Beijing Hospital/National Center of Gerontology of National Health Commission, Beijing, China; 3 Department of Stomatology, Beijing Chaoyang Hospital, Capital Medical University, Beijing, China

**Keywords:** Macrophage, Cardiomyocyte, Crosstalk, Macrophage phenotypes, Phagocytosis, Physical interaction, Paracrine signaling, Extracellular vesicles

## Abstract

The heart is a muscular organ that pumps blood throughout the body and is one of the most vital organs in human body. While cardiomyocytes are essential for maintaining the normal function of the heart, a variety of cardiovascular diseases such as coronary artery occlusion, arrhythmia, and myocarditis can lead to cardiomyocyte death, resulting in deterioration of heart function. The adult mammalian heart is incapable of regenerating sufficient cardiomyocytes following cardiac injuries, eventually leading to heart failure and death. Cardiac macrophages are ubiquitously distributed in the healthy heart and accumulated at the site of injury. Macrophages play essential roles in regulating homeostasis and proliferation of cardiomyocyte, promoting electrical conduction, and removing dead cardiomyocytes and debris through direct and indirect cell–cell crosstalk. In this review, we summarize the latest insights into the role of macrophages in maintaining cardiac homeostasis and the macrophage-cardiomyocyte crosstalk in both healthy and injured scenarios.

Video Abstract

**Supplementary Information:**

The online version contains supplementary material available at 10.1186/s12964-023-01202-4.

## Background

Cardiovascular diseases (CVDs) remain the leading cause of disease burden worldwide [1]. Heart failure is usually the end stage of many CVDs, with a five-year survival rate of less than 50%. Cardiomyocytes (CMs) are the main cellular components of the heart, and keep the heart functioning properly. However, adult CMs hardly regenerate and many of them will be lost after cardiac injuries such as ischemia, mechanical injury, and myocardial inflammation, eventually leading to cardiac fibrosis and heart failure.

With a spindle-shaped and elongated appearance, cardiac macrophage is the most abundant immune cell component that is ubiquitously distributed in the heart [2]. Providing immune defense and maintaining tissue homeostasis are the two fundamental functions of cardiac macrophages [3]. Pattern recognition receptors (PRR) expressed on the macrophages can recognize pathogen-associated molecular patterns (PAMPs) and damage-associated molecular patterns (DAMPs) and initiate the innate immune response [4]. According to their origins, macrophages can be classified into embryo-derived resident macrophages and recruited monocyte-derived macrophages [5]. In addition, macrophages can be activated into M1 or M2 subtype in response to different signals [6].

Beyond phagocytic function, macrophages have been regarded as one of the major cellular components of the heart as they are involved in electric activity [2], acute inflammatory response [7], and reparative processes [8] under steady state and pathological conditions. Specifically, each macrophage interacts with several CMs through various physiological and pathological processes [9]. Typically, macrophage-cardiomyocyte crosstalk can be achieved through physical interactions, paracrine signaling, or extracellular vesicles (EVs). This review aims to summarize recent findings on macrophage-CM interaction and communication during steady state and disorders and to discuss a therapeutic opportunity for CVDs.

## Characteristics of cardiac macrophages

### Macrophage phenotypes

Macrophages are an inherent part of cardiac tissue, where they are involved not only in maintaining normal cardiac homeostasis [2, 9] but also in resolving inflammatory responses and pathological progression [7, 10]. Importantly, cardiac macrophages are highly heterogeneous and dynamically change in response to environmental stimuli. In the normal heart, there are mostly resident macrophages and a few monocyte-derived macrophages [11]. After injury, CMs undergo necrosis and release DAMPs, which attract CCR2^+^ circulating monocytes. These monocytes and monocyte-derived macrophages remove dead cells and debris, secrete pro-inflammatory cytokines, and induce extracellular matrix degradation during the early stages of cardiac injury. Subsequently, a subset of resident macrophages secrete high levels of anti-inflammatory cytokines and facilitate tissue repair during inflammation resolution [12–14].

**Fig. 1:**
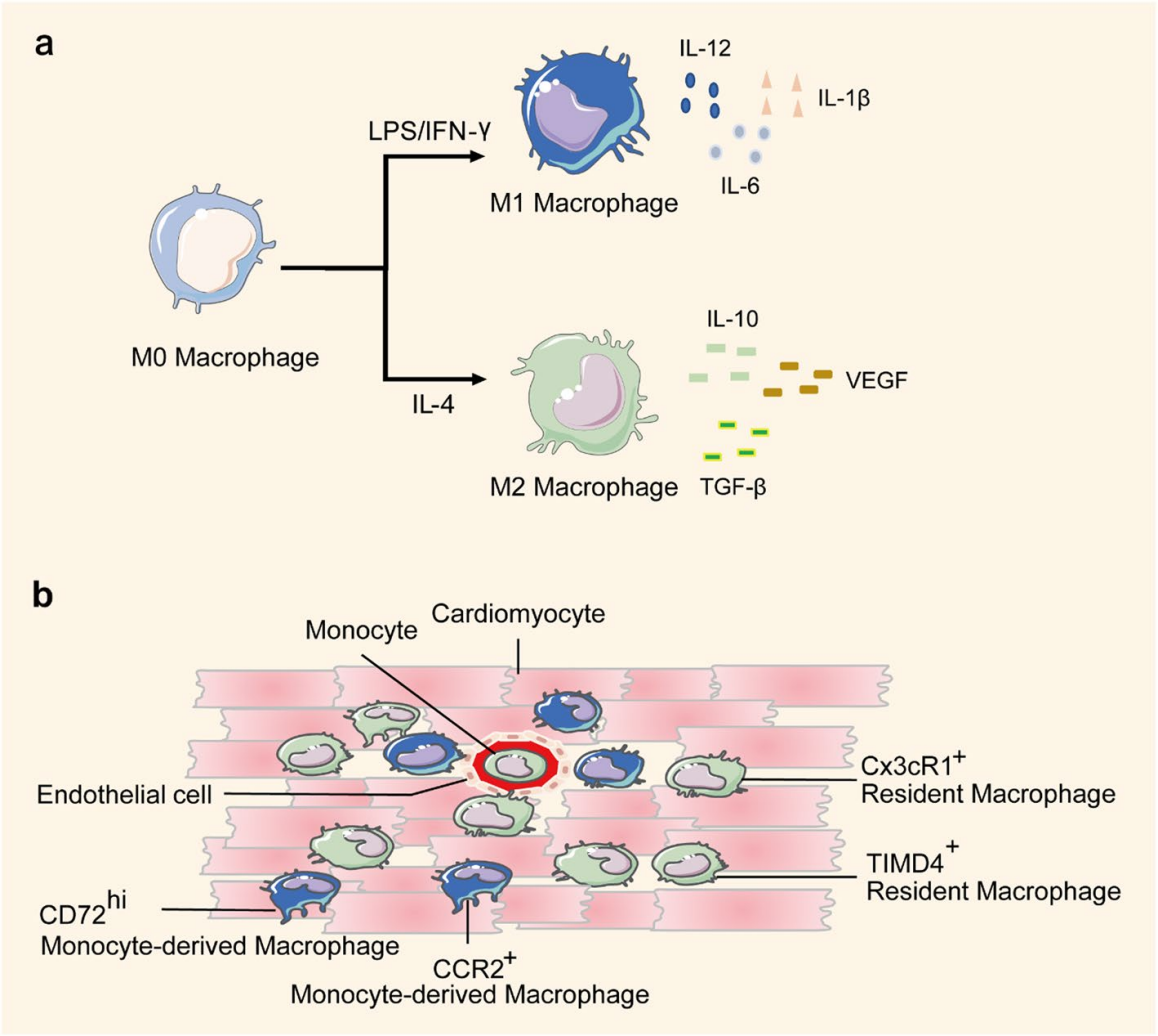
Macrophage Classification Modes. **a** M0 macrophages can be polarized into M1 macrophages by LPS/IFN-γ stimulation. M1 macrophages are pro-inflammatory subtypes that secrete pro-inflammatory cytokines such as IL-6, IL-12, and IL-1β. M0 macrophages can be polarized into M2 macrophages by IL-4 stimulation. M2 macrophages are associated with inflammation resolution and tissue repair through the secretion of anti-inflammatory cytokines such as IL-10, VEGF, and TGF-β. **b** Cardiac macrophages can be classified into CCR2^+^ monocyte-derived macrophage and CX3CR1^+^ resident macrophage according to their function and origins. Monocyte-derived macrophages are pro-inflammatory and lack reparative activities, whereas resident macrophages promote cardiac repair through anti-inflammatory activities, angiogenesis, and cardiomyocyte proliferation. Furthermore, TIMD4^+^ macrophages are regarded as a pure population of resident macrophages that reside in cardiac tissue. CD72^hi^ macrophages are pro-inflammatory macrophage subsets

Macrophages can be broadly classified as M1 or M2 subtypes (Fig. 1a). M1 macrophages are pro-inflammatory subsets that secrete pro-inflammatory cytokines such as interleukin-6 (IL-6), interleukin-12 (IL-12), and interleukin-1β (IL-1β), whereas M2 macrophages are associated with inflammation resolution and tissue repair by secreting anti-inflammatory cytokines such as interleukin-10 (IL-10), vascular endothelial growth factor (VEGF), and transforming growth factor-β (TGF-β) [6]. Some recent studies have attempted to facilitate cardiac recovery by inhibiting the inflammatory response of M1 macrophages or enhancing the repair activities of M2 subsets [15–17].

Another classification mode classifies macrophages according to their function and origin (Fig. 1b). CX3CR1^+^ macrophages are resident cardiac macrophages mainly derived from embryonic development, either from early erythromyeloid progenitors or from fetal monocytes [14]. After birth, CCR2^+^ macrophages from circulating monocytes, known as monocyte-derived macrophages, constantly infiltrate cardiac tissue [11]. After cardiac injury, these two macrophage subsets from different origins play opposite roles [8, 18, 19]. Monocyte-derived macrophages are pro-inflammatory and lack reparative activities. Selective inhibition of these macrophages enhances tissue repair by blocking monocyte recruitment and reducing inflammatory response [8, 20]. In contrast, embryonic-derived resident cardiac macrophages promote cardiac repair through anti-inflammatory activities, angiogenesis, and CM proliferation by secreting cytokines such as oncostatin M (OSM), myeloid-derived growth factor (Mydgf) and METRNL (meteorin-like) [8, 18, 19, 21]. Furthermore, recent studies have identified additional macrophage phenotypes with specialized functions using single-cell transcriptomics and fate mapping [22, 23]. For example, TIMD4^+^ macrophages were considered to be a pure population of resident macrophages residing in cardiac tissue [22]. Legumain (Lgmn) was a gene specifically expressed by cardiac resident macrophages that mediated efferocytosis after cardiac injury [23]. Using single-cell transcriptomic analyses of cardiac immune cells, Ni et al. identified monocyte-derived CD72^hi^ macrophages as a pro-inflammatory macrophage subset that mediated CM oxidative stress and apoptosis, which was similar to CCR2^+^ monocyte-derived macrophages [24]. Since the cardiac microenvironment is complicated and cardiac macrophages are dynamic, more detailed macrophage subsets and functions need to be further explored.

### Phagocytic function of macrophages

**Fig. 2:**
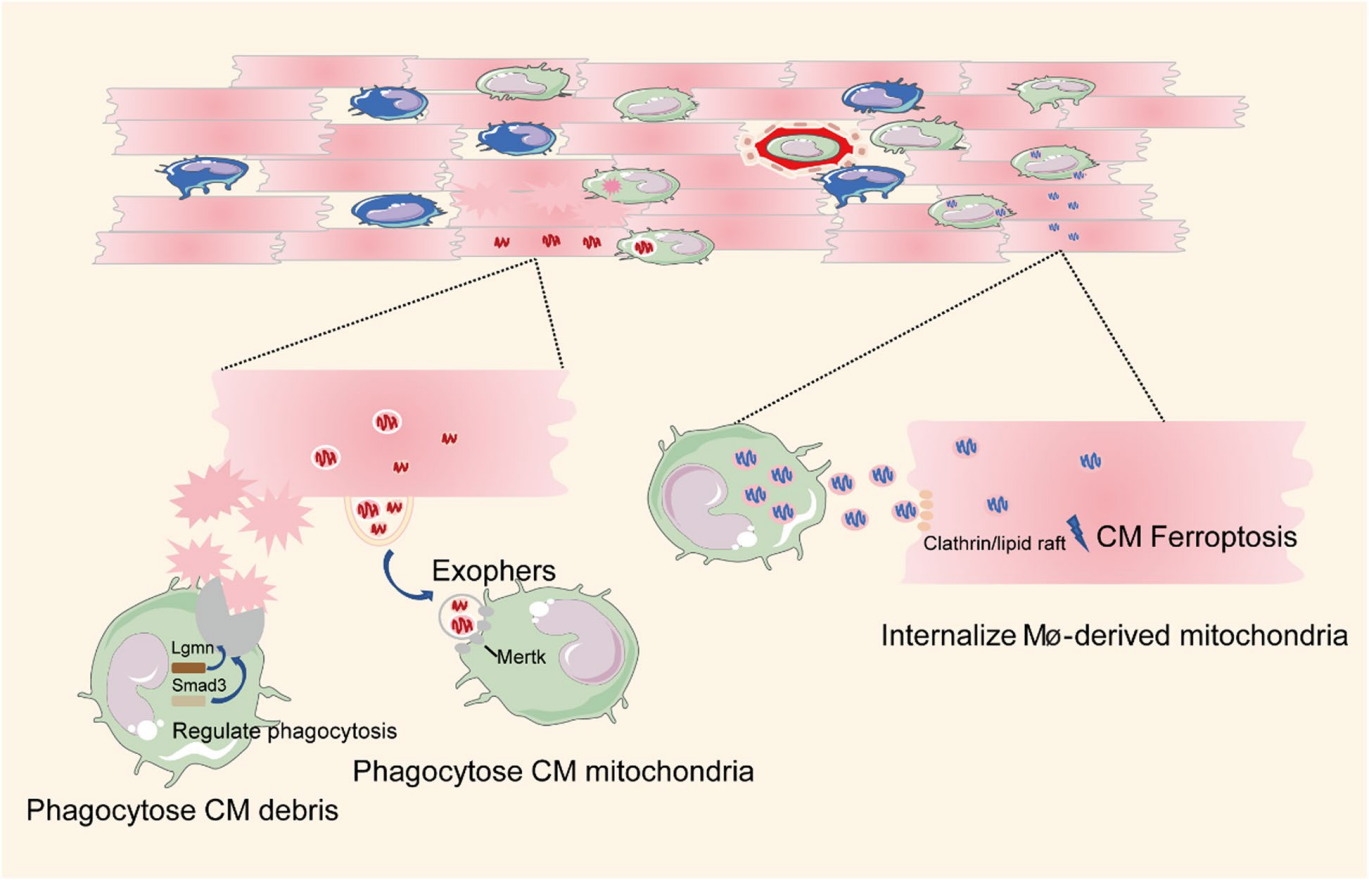
Phagocytic Function of Macrophages (Mø). Macrophages phagocytize CM debris. The phagocytic function of macrophages is regulated by Lgmn and Smad3 signaling. In addition, macrophages engulf dysfunctional mitochondria from CMs via MerTK. Similarly, CMs internalize macrophage-derived mitochondria by clathrin- or lipid raft-mediated endocytosis and induce CM injury by triggering ferroptosis

During the innate immune response, macrophages recognize PAMPs and DAMPs released by pathogens and damaged cells and exert effects of phagocytosis, complement activation, and cell apoptosis induction [4]. Cardiac macrophages remove necrotic and apoptotic CMs and metabolites during cardiac homeostasis and injury states (Fig. 2). In a recent study, CMs shed dysfunctional mitochondria and other cargos into dedicated membranous particles to maintain cardiac homeostasis, and these particles were termed “cardiac exophers” [9]. Macrophages engulfed the exophers through the phagocytic receptor mer tyrosine kinase (MerTK). Notably, depletion of macrophages prior to ischemia resulted in the accumulation of anomalous mitochondria in CMs, metabolic alterations, and ventricular dysfunction [9]. Similarly, a cardiomyocyte-macrophage co-culture experiment showed that CMs could internalize macrophage-derived mitochondria through clathrin- or lipid raft-mediated endocytosis, thereby inducing CM injury by triggering ferroptosis [25]. In a skeletal muscle regeneration study, macrophage-derived glutamine boosted satellite cell proliferation and muscle regeneration [26]. Therefore, there may be other metabolites in cardiomyocyte-macrophage interactions.

The mechanisms regulating macrophage phagocytosis are not fully understood. A recent study showed that Lgmn, which is specifically expressed in cardiac resident macrophages, was a potential candidate gene to regulate macrophages in the clearance of dying CMs [23]. Specifically, Lgmn deficiency severely inhibited the clearance and degradation of dying CMs by impairing macrophage efferocytosis pathways [23]. Conversely, selective overexpression of Lgmn in resident macrophages improved cardiac function after myocardial infarction, providing a therapeutic approach for cardiac repair [23]. Macrophage SMAD family member 3 (SMAD3) signaling also protected the infarcted heart by mediating phagocytosis of dead CMs [27]. Taken together, further characterization of the signals that regulate phagocytosis has the potential to provide meaningful therapeutic avenues for the failing heart.

## Crosstalk between macrophages and cardiomyocytes in physiological and pathological conditions

### Physical interaction

The myocardium is composed of various cell types, with CMs accounting for approximately one-third [12]. However, the population of macrophages is much smaller than that of CMs [2]. They are present in higher density in the ventricles and atrioventricular (AV) nodes [2, 9]. It has been reported that each CM is surrounded by an average of five macrophages [2, 9]. Notably, different macrophages are present in different ways. A recent study confirmed that CCR2^−^ macrophages physically contacted neighboring CMs, while CCR2^+^ macrophages did not directly contact CMs but extended their processes into the interstitial space [10]. In addition, it was reported that CCR2^+^ macrophages resided in the endocardial trabeculae, whereas CCR2^−^ macrophages remained in the compact myocardium [19]. Another study reported that CCR2^−^ macrophages were replenished through local proliferation, whereas CCR2^+^ macrophages were maintained by monocyte recruitment and proliferation [28]. These two macrophage subtypes may derive from diverse progenitor cells and localize within different regions of the embryonic heart, leading to their different modes of contact with CMs.

**Fig. 3:**
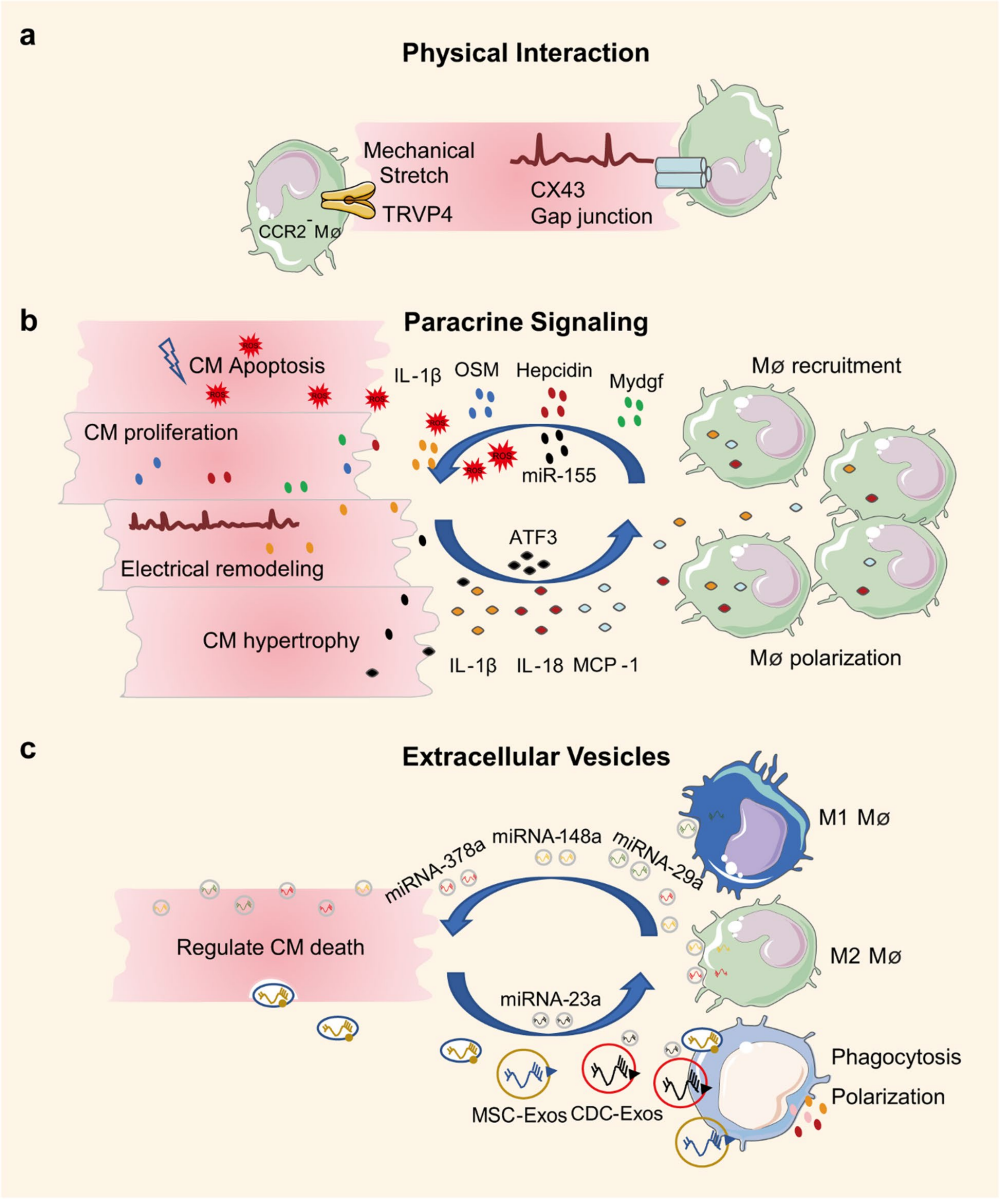
Modes of Communication between Macrophages and CMs. **a** Physical interaction. Macrophages interact with neighboring CMs through physical interaction. Macrophages mediate adaptive myocardial remodeling by sensing mechanical stretch of CMs and facilitate CMs electrical conduction by connexin 43. **b** Paracrine signaling. Macrophages secrete cytokines or other molecules such as IL-1β, OSM, Hepcidin, and Mydgf to regulate CM proliferation, hypertrophy, apoptosis, and electrical conduction. CMs also secrete cytokines such as IL-1β, IL-18, and MCP-1 to recruit macrophages and regulate the phagocytic function of macrophages. **c** Extracellular vesicles. Macrophages produce vesicles such as miRNA-378a, miRNA-148a, and miRNA-29a to regulate CM death, and CMs also secrete vesicles such as MSC-Exos and CDC-Exos to regulate macrophage polarization and phagocytosis

In a mouse model of dilated cardiomyopathy, CCR2^−^ macrophages interacted with neighboring CMs through focal adhesion complexes to promote left ventricle (LV) dilation and coronary dilation and to maintain adequate cardiac output. Mechanistically, these macrophages were activated in response to mechanical stretch through a transient receptor potential vanilloid 4 (TRPV4)-dependent pathway that controls the expression of growth factors [10]. This finding implicates mechanical sensing in cardiac macrophage activation under adaptive cardiac remodeling (Fig. 3a).

Normal electrical activity is fundamental to the regular beating of the heart, and the AV node plays an essential role in the electrical connection between the atrium and the ventricles. One study reported that macrophages interacted with CMs through connexin 43 and facilitated electrical conduction through the distal atrioventricular node [2] (Fig. 3a). Additionally, a recent study reported the actions of macrophages during right ventricular (RV) dysfunction [29]. They found that macrophage-derived amphiregulin (AREG) induced gap junctional intercellular communication between CMs by controlling connexin 43 phosphorylation and translocation via an EGFR/MEK/ERK pathway [29]. Conversely, macrophage ablation induced progressive atrioventricular block [2] and severe arrhythmia [29], suggesting macrophages are required for the communication and regulation of CMs. However, a similar study showed that arrhythmias or alterations in conduction could not be identified following CCR2^−^ macrophage depletion [10], suggesting further research is needed to identify how different macrophage populations regulate cardiac electrical activities.

### Paracrine signaling

Both CMs and macrophages exert paracrine effects by secreting various cytokines (Fig. 3b). CMs induce inflammatory gene expression and activate the NOD-like receptor thermal protein domain associated protein 3 (NLRP3) inflammasome following cardiac injury, and these responses elicit macrophage recruitment [30]. Previous research has revealed a direct cardiomyocyte-macrophage crosstalk in sepsis-associated cardiomyopathy [31]. It was found that unstimulated CMs maintained macrophage functional quiescence by secreting soluble inhibitors. Furthermore, when stimulated by group A streptococcus, CMs produced several stimulators that boosted the macrophage production of IL-6, IL-1β, nitric oxide synthase (iNOS), and matrix metalloproteinase (MMP9) [31]. Interestingly, a recent cell culture study confirmed that macrophage-derived sulfur dioxide (SO_2_) was involved in macrophage chemotaxis and activation [32]. Thus, the cell-derived SO_2_ may regulate the interactions among cardiac cells in addition to macrophages themselves.

In adult mammals, the loss of proliferation and renewal capacity of CMs prevents full regenerative repair and ultimately leads to heart failure. One promising approach to improve the prognosis of heart failure is to promote CM proliferation. It was reported in 2014 that acute inflammation characterized by macrophage recruitment is required for CM proliferation and cardiac regeneration in neonatal mice [7]. Macrophage-derived OSM is an important upstream cytokine in promoting CM proliferation and cardiac regeneration through the OSMR/gp130 heterodimer receptor [33]. And Mydgf is a paracrine protein secreted by monocytes and macrophages that promote CM proliferation and cardiac regeneration through the c-Myc/FoxM1 pathway [34]. In contrast, the presence of some substances (e.g., the iron regulator hepcidin) in macrophages refrained CM proliferation and cardiac repair by modulating the IL-4/IL-13 pathways [35].

Unlike adult mammals, zebrafish can fully regenerate an injured heart through CM proliferation [36]. A larval zebrafish cardiac injury study confirmed that macrophages were recruited to the epicardial-myocardial niche after cardiac injury and induced CM proliferation by regulating epicardial vascular endothelial growth factor aa (Vegfaa) and endocardial Notch signaling [37]. The identification of this macrophage-cardiomyocyte interaction mechanism highlights the important regulatory role of macrophage in CM proliferation.

Macrophages are highly heterogeneous. Although there is literature in this area, the specific subtypes of cardiac macrophages and their exact identities are not currently clear. What we do know is that the neonatal heart has a remarkable capacity for cardiac repair, whereas the adult heart does not [38], and that neonatal cardiac macrophages are quite different from adult macrophages [8]. Macrophage characterization and lineage tracing studies revealed that the neonatal heart contained an embryonic-derived lineage of CCR2^−^ macrophages that caused minimal inflammation and promoted coronary angiogenesis and CM proliferation after cardiac injury. The in vitro co-culture experiment also showed that only neonatal CCR2^−^ macrophages could promote CM proliferation [8]. Transplantation of murine neonatal cardiac embryonic-derived CX3CR1^+^ macrophages could improve adult CM proliferation and cardiac repair after myocardial infarction [18]. The underlying mechanism via which neonatal macrophages or CX3CR1^+^ macrophages mediate CM proliferation may involve the secretion of cytokines such as OSM, Mydgf, and IL-4 [33–35]. In contrast, the injured adult heart recruited mainly CCR2^+^ monocytes and macrophages, which had a reduced capacity to promote angiogenesis or CM proliferation and instead enhanced inflammatory response [8]. Selective inhibition of CCR2^+^ monocytes and macrophages in the adult heart could preserve embryonic-derived macrophages and enhance cardiac repair, suggesting a therapeutic effect by blocking monocyte recruitment and reducing the inflammatory response [8, 20].

Hypertension, cardiomyopathy, or other cardiovascular diseases cause cardiac pressure overload and hypertrophy. Sustained pressure overload induces cardiac remodeling and consequent heart failure [39]. In an angiotensin II (Ang II)-induced pressure-overloaded mouse model, pressure overload-induced cardiac infiltration of miR-155-expressing monocytes and macrophages was consistent with the finding in a human study, in which miR-155 expression was increased in hypertrophic patients [40]. Furthermore, miR-155 regulated cardiac monocyte and macrophage infiltration, CM hypertrophy, and heart failure. Inhibition of miR-155 protected against cardiac hypertrophy and heart failure. Mechanistically, miR-155 expressing in macrophages promoted CM hypertrophic growth targeting suppressor of cytokine signaling 1 (Socs1) and signal transducer and activator of transcription 3 (Stat3) [40]. Another study used an α1-adrenergic agonist phenylephrine (PE) to mimic chronic pressure overload and hypertrophic cardiac remodeling processes, and showed that chronic PE infusion induced activating transcription 3 (ATF3) expression in CMs, which resulted in macrophage activation and recruitment, and finally cardiac maladaptive remodeling. In addition, inhibition of ATF3 expression in either macrophages or CMs attenuated cardiac remodeling processes [41]. This study identified a unique ATF3-dependent cross-talk between macrophages and CMs during maladaptive cardiac remodeling. However, the interaction mechanism between macrophages and CMs remained unclear, and the authors did not identify whether recruited macrophages contributed to maladaptive cardiac remodeling by secreting various cytokines and chemokines. It also remains to be determined whether ATF3 regulates other cardiac cells during cardiac hypertrophy.

Atrial fibrillation (AF) is the most common type of cardiac arrhythmia. Evidence suggests that both inflammation and macrophages are involved in the development of AF [42, 43]. There is a functional cross-talk between atrial CMs and macrophages in AF. Specifically, AF increases macrophage infiltration into the atria and polarizes them to a pro-inflammatory subtype. On the other hand, pro-inflammatory macrophages further aggravate atrial electrical remodeling by secreting IL-1β and tumor necrosis factor-α (TNF-α). Mechanically, Il-1β inhibits the expression of quaking protein (QKI) and further downregulates L-type calcium currents (I Ca-L) in CMs [44]. These findings provide novel insights into AF therapy from the perspective of inflammatory macrophages.

In the injured heart, oxidative stress occurs in the myocardium and then massive CMs die from hypoxia [45]. Macrophage-derived reactive oxygen species (ROS) is a mediator in the pathogenesis of cardiac injury. Inhibiting ROS generation may attenuate CM death and restore heart function [46]. By using human pluripotent stem cell (hPSC)-derived CMs and macrophages co-culture system, Yang et al. found that macrophages could induce increased ROS production and apoptosis in CMs after exposure to severe acute respiratory syndrome coronavirus 2 (SARS-CoV-2), whereas tofacitinib (a JAK inhibitor) and ranolazine (an anti-anginal drug) protected CMs from macrophage-induced cardiotoxicity by decreasing ROS production [47]. Similar to the CCR2^+^ macrophage subset, CD72^hi^ macrophages, as a subset of pro-inflammatory macrophages can induce oxidative stress and apoptosis in CMs after cardiac injury. In addition, Rel is the upstream transcription factor that induces myeloid cells to differentiate into CD72^hi^ macrophages. Although CD72 cannot be used in knockout animal models due to its broad expression in various immune cells, Rel knockout may decrease the levels of ROS and inflammatory cytokines, inhibit CM apoptosis, and ultimately mitigate cardiac injury. Thus, Rel may serve as a therapeutic target for CVDs [24].

### Extracellular vesicles (EVs)

EVs are derivatives of cell debris released from various cell types and act as information carriers. EVs can be divided into exosomes, microvesicles, and apoptotic bodies according to their different types of cellular or intracellular origin [48]. EVs are key mediators of intercellular communication in the cardiac microenvironment by exchanging cellular substances, such as microRNAs (miRNAs).

Macrophage-derived exosomes have specific effects on CM death (Fig. 3c). For example, macrophage-derived exosomes such as microRNA-29a (miRNA-29a) mediate CM pyroptosis [49], whereas M2 macrophage-derived exosomes such as microRNA-148a (miR-NA-148a) [50] and microRNA-378a-3p (miRNA-378a-3p) [51] reduce CM apoptosis and pyroptosis after cardiac injury. In addition, chemotherapeutic drugs such as programmed cell death 1 (PD-1) inhibitors are cardiotoxic. Xia et al. reported that PD-1 inhibitor-treated macrophages had a pro-senescent effect on CMs through the microRNA-34a-5p/PNUTS signaling pathway and induced cardiac senescence-related injury [52].

CM-derived vesicles can modulate macrophage polarization in a specialized profile, including increasing the expressions of iNOS, IL‐1β, and IL‐6, and regulating their phagocytosis and adhesion [53–55] (Fig. 3c). A co-culture experiment showed that Ang II-treated atrial myocytes could secrete mi-croRNA-23a (miRNA-23a), which inhibited macrophage M2 polarization. Furthermore, inhibiting the expression of microRNA-23a in atrial myocytes suppressed atrial fibroblast activation by promoting M2 macrophage polarization [56].

Exosomes from other cell types also regulate the phagocytosis and repair of macrophages (Fig. 3c). Mesenchymal stem cell-derived exosomes (MSC-exos) can enhance the opsonization of dead CMs and activate the phagocytic and pro-reparative signaling of macrophages, thereby promoting cardiac recovery after injury [57]. Furthermore, cardiosphere-derived cell secreted exosomes (CDC-exos) confers cardioprotective effects after cardiac injury by polarizing monocytes and macrophages towards reparative phenotypes and enhancing their phagocytic capacity through the key component microRNA-181b [58].

Notably, most of these studies have been conducted in vitro, and therefore additional in vivo experiments are needed to explore the effects of vesicles between CMs and macrophages. Overall, as cardiac macrophages are heterogeneous, further research is needed to characterize the different roles of EVs among different macrophage subtypes.

## Therapeutic implications

Studies on the communications between macrophages and CMs provide a mechanistic basis for the progression of CVDs. It is currently known that the acute immune response underlies the benefit of cardiac therapy [59, 60]. For example, the sodium-glucose co-transporter 2 (SGLT2) inhibitor empagliflozin displayed a cardio-protective effect by stimulating macrophages into anti-inflammatory subsets [61]. Some biomaterials, such as melanin nanoparticles (MNPs)/alginate (Alg) hydrogels, promoted cardiac repair by scavenging ROS to inhibit oxidative stress-induced CM damage and by targeting macrophage polarization [62]. Interleukin-7 (IL‐7), mainly produced by thymic stromal cells, aggravated myocardial ischemia/reperfusion (I/R) injury by promoting CM apoptosis through the regulation of macrophage M1 polarization and chemotaxis [63]. Accordingly, inhibition of IL-7 had a therapeutic effect [63]. Furthermore, the rapid clearance of MSC-derived EVs by the mononuclear macrophage system inhibited the protective effect of EVs, whereas EVs modified with membrane protein CD47 and anti-apoptotic miRNA-21a efficiently improved the biodistribution of EVs in the heart and further promoted cardiac repair after myocardial I/R injury [64].

The stimulator of interferon genes (STING) signaling pathway is a key signal transduction molecule in the innate immune response. STING deficiency or inhibition of STING signaling can alleviate monocyte and macrophage infiltration and reverse cardiac fibrosis after cardiac hypertrophy [65, 66]. Likewise, targeting CMs and inhibiting inflammation signaling in CMs are also important therapeutic directions. For example, sucrose‐nonfermenting-related kinase (SNRK) was a CM‐specific repressor that inhibited macrophage recruitment and cardiac inflammation [67]. Thus, targeting SNRK in CMs may be a therapeutic strategy for cardiac repair.

## Conclusions

In this article, we summarize the modes of interactions between macrophages and CMs and divide them into three categories: physical interactions, paracrine signaling, and extracellular vesicles. In particular, direct physical interaction is the main mode of communication between macrophages and CMs. Macrophages secrete cytokines and other molecules to regulate CM proliferation, hypertrophy, and electrical conduction, whereas CMs secrete cytokines to recruit macrophages and regulate the phagocytic function of macrophages. Finally, both macrophages and CMs produce different types of vesicles to regulate their functions.

Cardiac immune cells are diverse, with complex cell–cell interactions. The impact of immune cells and cardiac cell interactions, especially macrophages and CMs, on the regulation of cardiac function may be a future research direction. The emergence of multi-omics technologies, including transcriptomics, single-cell RNA sequencing, spatial transcriptomics, proteomics, and metabolomics, makes it possible to precisely resolve the information in cardiac tissue at the DNA, transcriptional, protein, and metabolic levels.

Although there is a wealth of basic research targeting immune inflammation and myocardial injury, few clinically translatable drugs or treatments have been available. Moreover, macrophages are highly heterogeneous and can dynamically change. A better understanding of the typologies and functions of different macrophage subsets will enable us to precisely target specific macrophage subtypes that regulate cardiac function, thus providing opportunities for optimized CVD treatment.

## Data Availability

Not applicable.

## Abbreviations

CVDs: Cardiovascular diseases
CM: Cardiomyocyte
PRR: Pattern recognition receptors
PAMPs: Pathogen-associated molecular patterns
DAMPs: Damage-associated molecular patterns
EV: Extracellular vesicles
IL-6: Interleukin-6
IL-12: Interleukin-12
IL-1β: Interleukin-1β
IL-10: Interleukin-10
VEGF: Vascular endothelial growth factor
TGF-β: Transforming growth factor-β
Lgmn: Legumain
MerTK: Mer tyrosine kinase
SMAD3: SMAD family member 3
Mø: Macrophage
AV node: Atrioventricular node
LV: Left ventricle
TRPV4: Transient receptor potential vanilloid 4
RV: Right ventricular
AREG: Amphiregulin
NLRP3: NOD-like receptor thermal protein domain associated protein 3
iNOs: Nitric oxide synthase
MMP9: Matrix metalloproteinase
SO2: Sulfur dioxide
OSM: Oncostatin M
Mydgf: Myeloid-derived growth factor
Vegfaa: Vascular endothelial growth factor aa
Ang II: Angiotensin II
Socs1: Suppressor of cytokine signaling 1
Stat3: Signal transducer and activator of transcription 3
PE: Phenylephrine
ATF3: Activating transcription 3
AF: Atrial fibrillation
TNF-α: Tumor necrosis factor-α
QKI: Quaking protein
I Ca-L: L-type calcium currents
miRNAs: MicroRNAs
miRNA-29a: MicroRNA-29a
miR-NA-148a: MicroRNA-148a
miRNA-378a-3p: MicroRNA-378a-3p
PD-1: Programmed cell death-1
miRNA-23a: Mi-croRNA-23a
MSC-exos: Mesenchymal stem cell-derived exosomes
CDC-exos: Cardiosphere-derived cell secreted exosomes
ROS: Reactive oxygen species
hPSC: Human pluripotent stem cell
SARS-CoV-2: Severe acute respiratory syndrome coronavirus 2
SGLT2: Sodium-glucose co-transporter 2
MNPs: Melanin nanoparticles
Alg: Alginate
IL‐7: Interleukin-7
I/R: Ischemia/reperfusion
STING: Stimulator of interferon genes
SNRK: Sucrose‐nonfermenting–related kinase
